# Influence of First-Time Mothers' Early Employment on Severe Early Childhood Caries in Their Child

**DOI:** 10.1155/2012/820680

**Published:** 2012-10-11

**Authors:** Kamila Plutzer, Marc J. N. C. Keirse

**Affiliations:** ^1^Australian Research Centre for Population Oral Health, School of Dentistry, The University of Adelaide, Adelaide, SA 5005, Australia; ^2^Department of Obstetrics, Gynaecology and Reproductive Medicine, Flinders Medical Centre, Flinders University, Bedford Park, SA 5042, Australia

## Abstract

*Aim*. To examine whether mothers' early employment status is related to the development of severe early childhood caries in their child. *Methods*. Questionnaire survey of 429 first-time mothers in metropolitan Adelaide, South Australia, and dental examinations of their child at 20 months of age. *Results*. At 20 ± 2.5 months of age, 5.6% of children exhibited caries defined as one or more demineralized or cavitated lesions on the upper incisors. Of the mothers, 52.2% had no paid employment, 39.6% were part-time and 8.2% full-time employed. Overall, mothers' participation in the workforce had no influence on the frequency of severe early childhood caries in their child, but there was a significant interaction with family structure. For mothers without employment there was no difference between single, and two-parent families, but children with an employed single mother more frequently had caries than those with a working mother in a two-parent family (*P* < 0.04). However, there were no significant differences in children's reported general health. *Conclusions*. The data indicate a need to explore strategies that may assist single mothers and especially those in the workforce to prevent severe early childhood caries in their child.

## 1. Introduction

Early childhood caries has been on the increase in many countries in recent years and has become a significant public health problem across the globe [1]. The USA National Institutes of Health defined early childhood caries as one or more decayed (cavitated or noncavitated), missing because of caries, or filled primary teeth in a child under 6 years of age [2]. In children younger than 3 years of age, any sign of smooth surface caries (cavitated or not) is considered to be severe early childhood caries [2]. Early childhood caries seriously affects a child's well-being, learning ability, and quality of life [3]. It also impacts on the quality of life of its parents [3]. Moreover, early childhood caries has consequences even after the primary teeth have exfoliated. It is known to be the most powerful predictor of caries in the subsequent permanent teeth [4, 5] with all its ill effects, financial, and otherwise for the individual and the health-care system. 

 A review of epidemiological data since 2000 in a range of low-, middle- and high-income countries, indicated a marked increase in the prevalence of caries in both the deciduous and permanent dentitions of young children [1]. In the 1990s, the prevalence of severe early childhood caries in 2-3 years old children in South Australia was already 16.7% [6]. In Australia as a whole, the mean dmft (the average sum of decayed, missing due to caries, and filled teeth) at 4 years of age, the youngest group for which oral health data are available, increased from 1.1 in 1997 to 1.7 in 2004. In 2004, 38 percent of 4-year-old children had at least one tooth decayed, filled, or absent due to caries [7]. For some time now, early childhood caries has been the main reason for hospitalization of children aged 4 years or less in Australia [8], nearly all of whom require general anesthesia to address the problem. 

 No single factor has been identified to be responsible for the increased prevalence of early childhood caries, which is an infectious and communicable disease that originates from an interaction among several biological, behavioral, and social determinants. Traditionally, research in this area has focused mainly on biological and dietary factors. In recent years, the focus has broadened. Social, economic, and environmental factors are now recognized to be significant contributors to the development of the disease. This applies in Australia too where the prevalence of early childhood caries is substantially higher in rural than in metropolitan areas [9] and especially high in its Aboriginal population [10]. 

 Many studies have indicated that early childhood caries is largely preventable by good oral hygiene of mothers and children and proper eating habits instituted by mothers early on [11–15]. However, mothers of young children have several competing tasks. Not the least of these is joining the workforce to support the family budget, to advance or resume their professional career or for any other reasons. We found that providing mothers with information on caries prevention reduced the frequency of severe early childhood caries fivefold from 9.6% to 1.7% [12]. In absolute, but not in relative, terms the effect was greater in children from one-parent families than in those from two-parent families [16]. We, therefore, sought to examine whether mothers' early return to work would have an influence on the development of severe early childhood caries in their child compared with stay-at-home mothers. 

## 2. Methods

Data for this study were derived from a randomized controlled trial which enrolled 649 pregnant women expecting their first child in an oral health promotion intervention programme “Cavity-free children” with the aim of reducing the prevalence of early childhood caries at 1.5 years of age [12]. They were enrolled during routine antenatal visits at all 5 public maternity hospitals in metropolitan Adelaide and represented 20 percent of all women with a first singleton pregnancy in public hospitals in Adelaide, South Australia. Details of the trial have been published previously [12]. Women in the intervention group received three rounds of printed information on oral health maintenance, emphasising the importance of mothers' practices, and the specific health needs of their child at that particular stage of its development (obtainable from the authors). The first round, focusing on mothers' oral health, was provided at enrolment in the study, the second when the child was 6 months old, and the third when the child was 12 months old. The latest two sets were mailed to the home address. There was no interaction with the control group between enrolment and the outcome assessment at 20 months. 

Twenty-five mothers were excluded due to the absence of a mother-infant pair at followup or a congenital abnormality in the child, and 441 brought their child for an assessment of their child's dental status at 20 ± 2.5 (SD) months of age. Of these mothers, 429 had completed a questionnaire providing demographic details on their employment status, family structure (single, or two-parent family), and level of education. Levels of education were defined on the basis of the highest diploma or certificate obtained. Thus, mothers with incomplete secondary education were classified as having completed only primary education, and mothers with only some higher education were classified as having completed only secondary education. 

Questionnaires were completed by mothers in the privacy of their home before attending the examination. Mothers also answered questions about their child's general health and whether the child had ever been hospitalized after being taken home after birth. We did not explore the reasons why mothers entered or reentered the workforce after giving birth or why they had not done so. 

All dental examinations were conducted by the same dentist who had been independently calibrated in the conduct of these examinations, and was blinded to the mothers' characteristics. Children were examined sitting on their mother's lap following a standard protocol for the dental examination of young children [12]. The study focused on the earliest manifestation of severe early childhood caries, defining it as the presence of any cavitated or non cavitated lesions on the upper incisors, which are readily visible without the use of sharp instruments. 

Statistical analyses including chi-square tests for categorical variables and logistic regression analyses were conducted with PASW Statistics 18.

Approval for the study was obtained from the ethics committees at all 5 hospitals where women were recruited and from the Human Research Ethics Committee at the University of Adelaide. All mothers signed informed consent.

## 3. Results

Demographic characteristics of the mothers in the study are shown in Table 1. Their age distribution mimicked that of all mothers giving birth to a first child in South Australia. At the time of the examination of their child at 20 ± 2.5 months of age, 47.8% were either self-employed or in paid employment, but only 17% of them full time. Their age distribution was similar to that of stay-at-home mothers, but they were more likely to be part of a two-parent family (85.3% versus 76.2%; *P* < 0.03), and to have completed tertiary education (32.1% versus 10.5%; *P* < 0.02) than stay-at-home mothers. 

Table 2 shows that a third of working mothers (32.7%) resumed work within 6 months after giving birth, but only 8% did so within the first 3 months, with no difference between those in part-time or full-time occupations. About 60 percent relied on a grandparent, mostly the grandmother, and 38 percent relied on the child's father to look after their child during their absence (Table 3). Forty percent of working mothers relied on at least two child carers (Table 3).

 Mothers' opinions on the general health of their child and whether the child had ever been hospitalized after being taken home after birth are shown in Table 4. Twenty-eight percent of children had spent at least one day in hospital for one reason or another after having left hospital as a neonate. None of them were reported to be currently in ill health, though. Table 4 also shows the frequency of early childhood dental caries at 20 ± 2.5 months of age. Just over 1 in 20 (5.6%) showed signs of severe early childhood caries at this early age. However, none of the indices of child health differed between the children of employed and stay-at-home mothers (Table 4). There was a significant interaction, though, with family structure. For mothers without employment, there was no difference between single, and two-parent families, but children with a working single mother more frequently exhibited caries than those with a working mother in a two parent family (*P* < 0.04).

 Logistic regression analysis, shown in Table 5, indicates that, at this early age, caries prevalence increased with about 20% for each extra month of age. It also indicates that, on its own, mothers' employment or early return to work had little influence on the development of early childhood caries in their child (Table 5). Having only one parent or two parents seems to be of much greater importance. 

## 4. Discussion

Mothers are the gatekeepers to the health and healthy behaviors of their children [17–19]. However, mothers' role in society has changed drastically from the traditional model of caring for children and looking after their husband's needs. Currently, women comprise 46 percent of the workforce in Australia with 54 percent of them working full time. In 1979, when maternity leave, unpaid at the time, was introduced in Australia, few mothers returned to work before their child reached school age. In 2011, 35 percent of mothers had returned to work by the time their child reached one year of age and 50 percent were back at work by the time the child was 2 years old. Paid maternity leave was only introduced in Australia in 2011 and still applies only to the public sector, although private companies are increasingly providing paid maternity leave in order to attract staff, maintain an experienced workforce, and offset the costs of training new employees. Over that period, family structure changed too. In the 2006 Australian census, 22 percent of families were single-parent families (Australian Bureau of Statistics, available on http://www.abs.gov.au/AUSSTATS) roughly similar to that seen in our study population. 

 In our randomized controlled trial of providing new mothers with comprehensive guidance on preventing early childhood caries, we found that the benefits of the intervention were much greater in absolute terms for children in single-parent families than in two-parent families. This was not so in terms of relative risk reduction, though. The intervention reduced the frequency of early childhood caries only 3.5-fold (from 16.3% to 4.5%) in single-mother families compared with 7-fold (from 8.1% to 1.1%) in two-parent families [16].

 Although we had anticipated that mothers' early return to work would have an influence on the development of early childhood caries in their child, there was no evidence to that effect. The only effect that was sustained in univariable and multivariable analyses was single parenthood. Obviously, single mothers do not fit into one category. Some may have been divorced, separated, widowed, or single by choice. Nonetheless, our data seem to indicate that single mothers are less effective in maintaining the oral health of their child than other mothers, especially if they also have occupational obligations. By itself, mothers' return to work does not seem to be an issue in our community, but there is much to be said for providing extra support to mothers and children living in a single-parent family or at least to explore how their relative disadvantage can be counteracted. After all, what applies to severe early childhood caries may apply to other health issues too. 

## Figures and Tables

**Table 1:** Characteristics of mothers in the study.

Mothers' characteristics	Employed		Unemployed		Total	
	No.	%	No.	%	No.	%
Employment status					429	100.0
Full time	35	17.1	0	—	35	8.2
Part time	170	82.9	0	—	170	34.6
Unemployed	0	—	224	100.0	224	52.2
Maternal age at childbirth (years)						
Under 25	80	39.0	106	47.3	186	43.4
25–29	73	35.6	68	30.4	141	32.9
30–45	52	25.4	50	22.3	102	23.8
Mother's highest completed education						
Primary	55	28.1	83	37.9	138	33.3*
Secondary	78	39.8	91	41.6	169	40.7*
Tertiary	63	32.1	45	10.5	108	26.0*
Not disclosed	9		5		14	
Family status						
Single parent	29	14.7	50	23.8	79	19.4**
Two parents	168	85.3	160	76.2	328	80.6**
Not disclosed	8		14		22	

^∗^ *P* < 0.02; ^∗∗^ *P* < 0.03.

**Table 2:** Timing of mothers' return to the workforce.

Child's age at mother's return to work	Full time		Part time		Total	
	No.	%	No.	%	No.	%
Less or equal to 3 months	2	5.8	15	8.8	17	8.3
Between 3 and 6 months	12	34.3	38	22.4	50	24.4
After 6 months up to 1 year	18	51.4	94	55.3	112	54.6
From 1 to 2 years	3	8.5	23	13.5	26	12.7

Total	35	100.0	170	100.0	205	100

**Table 3:** Usual carer for the child during mother's absence at work*.

Usual carer	Number	Percent of children*	Percent of carers*
Grandmother	107	52.2	37.8
Father	77	37.6	26.0
Child care centre	53	25.9	17.9
Grandfather	24	11.7	8.1
Family child care**	14	6.8	4.7
Other family member	11	5.4	3.7
Other	10	4.9	3.4

Total	296	—*	100.0

*Of the 205 children, 123 (60%) had one usual carer, 67 (32.7%) had two carers and 15 (7.3%) had three. **Family child care refers to a child cared for by a foster mother in her home.

**Table 4:** Characteristics of the children.

Mothers' employment*	Employed		Unemployed		Total	
Child's characteristics*	No.	%	No.	%	No.	%
Gender						
Male	105	51.2	115	51.3	220	51.3
Female	100	48.8	109	48.7	209	48.7
Hospitalized since discharge from hospital						
No	145	74.0	156	70.6	301	72.2
Yes	51	26.0	65	29.4	116	27.8
Not disclosed	9		3		12	
General health as reported by mother						
Excellent	99	48.3	120	53.8	219	51.2
Very good	84	41.0	70	31.4	154	36.0
Good	22	10.7	33	14.8	55	12.9
Not disclosed	0		1		1	
Caries on dental examination						
No	194	94.6	211	94.2	405	94.4
Yes	11	5.4	13	5.8	24	5.6

*None of the data show a statistically significant difference between mothers' employment status.

**Table 5:** Effect of various factors on the prevalence of severe early childhood caries at 20 ± 2.5 months of age in univariable and multivariable logistic regression analyses*.

Variable	Univariable analysis		Multivariable analysis*	
	OR	95% CI**	OR	95% CI**
Child's gender				
Male	reference		reference	
Female	0.45	0.19–1.11	0.48	0.19–1.18
Age at dental exam (months)	**1.18**	**1.03–1.36** ^†^	**1.20**	**1.02–1.37** ^†^
Family structure				
One parent	reference		reference	
Two parents	**0.37**	**0.16–0.88** ^†^	**0.37**	**0.14–0.93** ^†^
Mother's age at childbirth in years				
Under 25	reference		reference	
25 to 29	0.84	0.32–2.24	0.84	0.27–2.65
30 to 45	0.59	0.19–1.84	0.55	0.17–1.75
Mother's highest level of education				
Primary	reference		reference	
Secondary	0.66	0.22–2.04	0.47	0.12–1.81
Tertiary	0.89	0.33–2.48	0.87	0.29–2.57
Mother's' employment				
Employed	reference		reference	
Unemployed	1.09	0.47–2.48	0.97	0.41–2.30

*Variables entered in the multivariable analyses were age at examination in months, age at first tooth eruption in months, child's gender, mother's age, level of education and employment status.

**OR: odds ratio; 95% CI: 95% confidence interval.

^†^ *P* < 0.01.
